# Clozapine-Induced Acute Pericarditis and Cardiac Tamponade: Early Recognition Improves Clinical Outcomes

**DOI:** 10.7759/cureus.79927

**Published:** 2025-03-02

**Authors:** Muhammad Waqar Elahi, David Shi, Eric Huang, Bharath Bhushan

**Affiliations:** 1 Medicine, West Virginia University School of Medicine, Morgantown, USA; 2 Internal Medicine, West Virginia University School of Medicine, Morgantown, USA

**Keywords:** acute pericardial effusion, anti-psychotic drug, cardiac tamponade, clozapine side effects, pericarditis, treatment resistant schizophrenia

## Abstract

Clozapine is approved for treatment-resistant schizophrenia. Many cardiovascular side effects are associated with clozapine, including QTc prolongation and myocarditis, which is a black box warning. Few cases of pericarditis have been reported in recent literature. We report a case of a 26-year-old male with schizoaffective disorder who presented with sharp chest pain and shortness of breath. The patient was started on clozapine three months prior to his current presentation. The workup revealed a large pericardial effusion with cardiac tamponade physiology and signs consistent with acute pericarditis. He required emergent pericardiocentesis and pericardial drain placement. Symptoms resolved after treatment with ibuprofen and colchicine. This case report highlights the importance of early diagnosis of life-threatening cardiovascular complications associated with clozapine, especially acute pericarditis and cardiac tamponade due to pericardial effusion. We strongly recommend implementing monitoring protocols to prevent this potentially life-threatening adverse effect in patients taking clozapine.

## Introduction

Clozapine is a second-generation antipsychotic approved for treatment-resistant schizophrenia in patients who fail to respond to typical antipsychotics. It has proven efficacy in reducing suicidality among schizophrenic patients, improving drug-induced tardive dyskinesia, reducing aggressive behavior, and treating both positive and negative schizophrenia symptoms [1-3]. Despite its effectiveness, its use has been limited due to serious adverse effects. The most common and severe side effects include agranulocytosis, respiratory depression, and seizures [4]. Cardiovascular side effects include cardiomyopathy, QTc prolongation, myocarditis, and rarely pericarditis [5]. Although very rare (>1/10000), cardiovascular side effects can be life-threatening, and hence, early recognition and treatment are crucial. The symptoms of clozapine-induced pericarditis may have a variable presentation and insidious onset. Symptoms may include shortness of breath, chest pain, tachycardia, heart palpitations, and fatigue [6]. These symptoms may often be confused with psychiatric manifestations, including anxiety, panic attacks, or somatization, which can delay the accurate diagnosis and can lead to life-threatening complications [7].

## Case presentation

A 26-year-old male patient with a history of schizoaffective disorder, bipolar type; catatonia; a cerebrovascular accident at age 15 with residual right-sided weakness; and seizure disorder presented to the hospital with a three-month history of progressively worsening shortness of breath and intermittent sharp chest pain with radiation to the back.

The patient was recently hospitalized in a psychiatric facility approximately four months ago, where he was started on clozapine due to worsening psychosis. He was initially started on clozapine 12.5 mg daily and titrated up to 250 mg daily before discharge from the psychiatric facility. He started experiencing sharp chest pain and shortness of breath one week after his discharge. These symptoms were intermittent and not severe enough to warrant an emergency department (ED) visit. 

**Figure 1:**
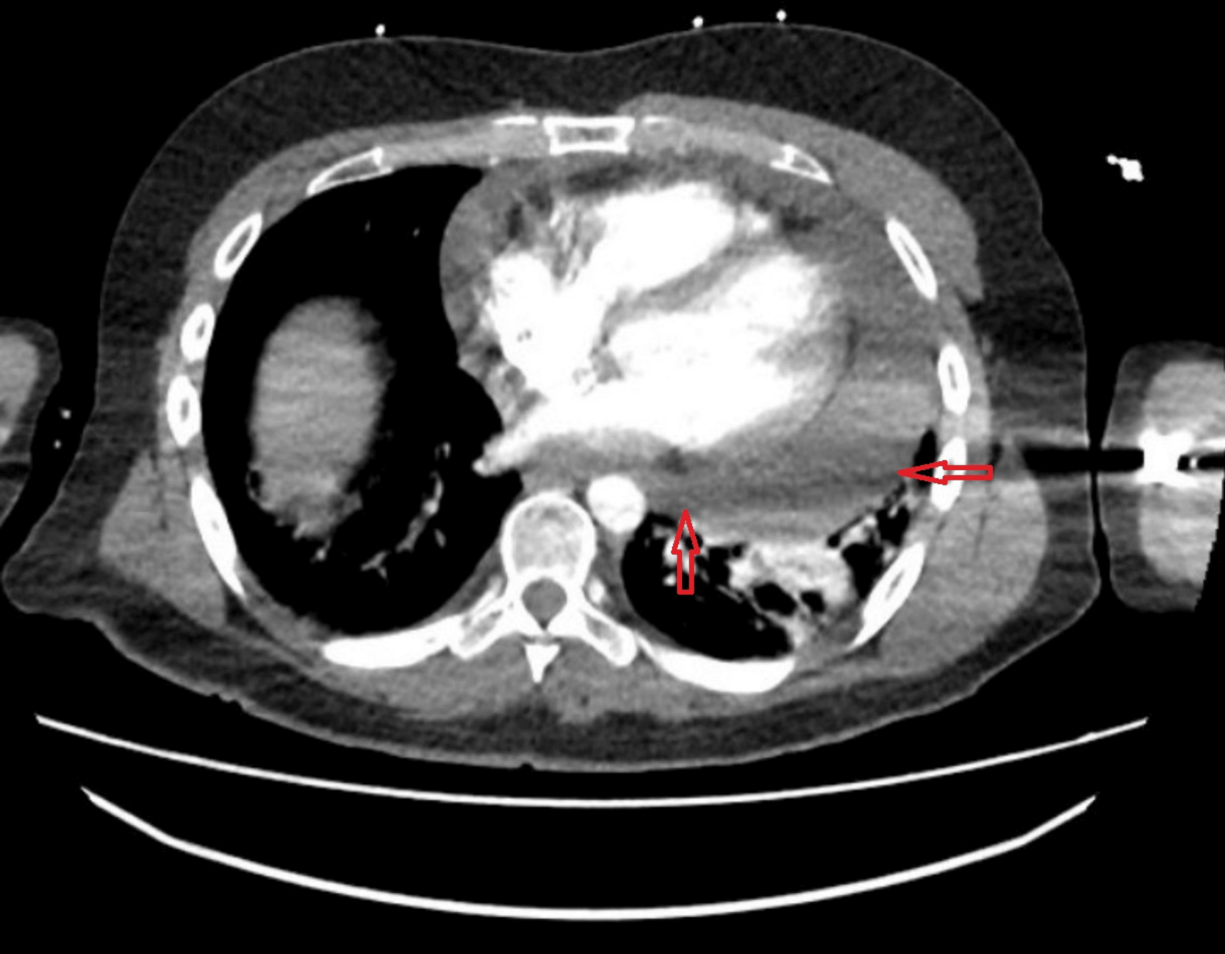
CT angiography chest showing a large pericardial effusion. CT: Computed tomography

**Figure 2:**
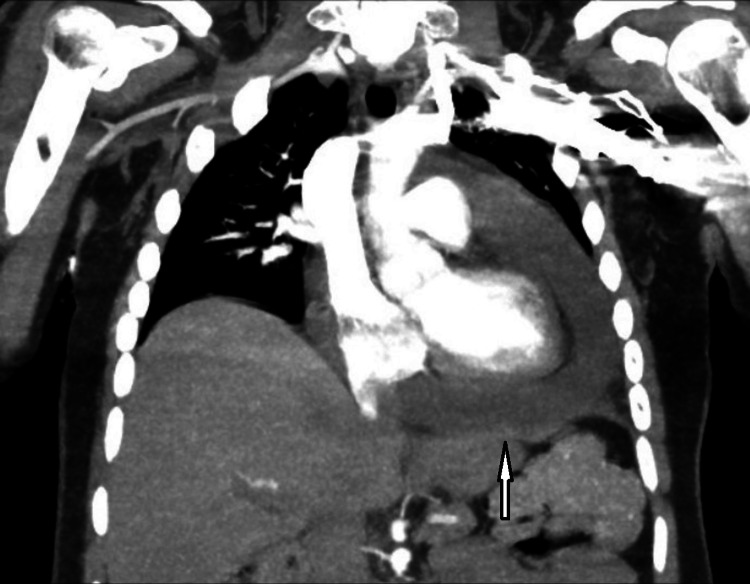
CT angiography chest showing a large pericardial effusion, which is concerning for a cardiac tamponade. CT: Computed tomography

In our ED, the patient was tachycardic with a heart rate in the 120s/min, blood pressure of 143/70 mm Hg, oxygen saturation at 94% on room air, and was afebrile. Laboratory results, including troponin, B-type natriuretic peptide (BNP), complete blood count (CBC), basic metabolic panel (BMP), and hepatic panel, were within normal range except for mildly elevated C-reactive protein (CRP) of 32 mg/L (reference range: < 8 mg/L). Chest computed tomography (CT) angiography showed a large pericardial effusion with some pulmonary edema (Figures 1, 2).

**Figure 3:**
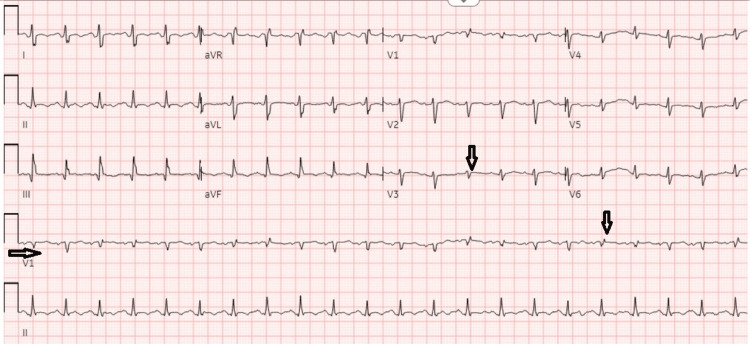
EKG showing sinus tachycardia with low voltage QRS complexes (arrows). EKG: Electrocardiogram Low voltage QRS complexes are more prominent in V1 & V3.

An electrocardiogram (EKG) was done, which showed sinus tachycardia with new-onset low voltage (Figure 3).

**Figure 4:**
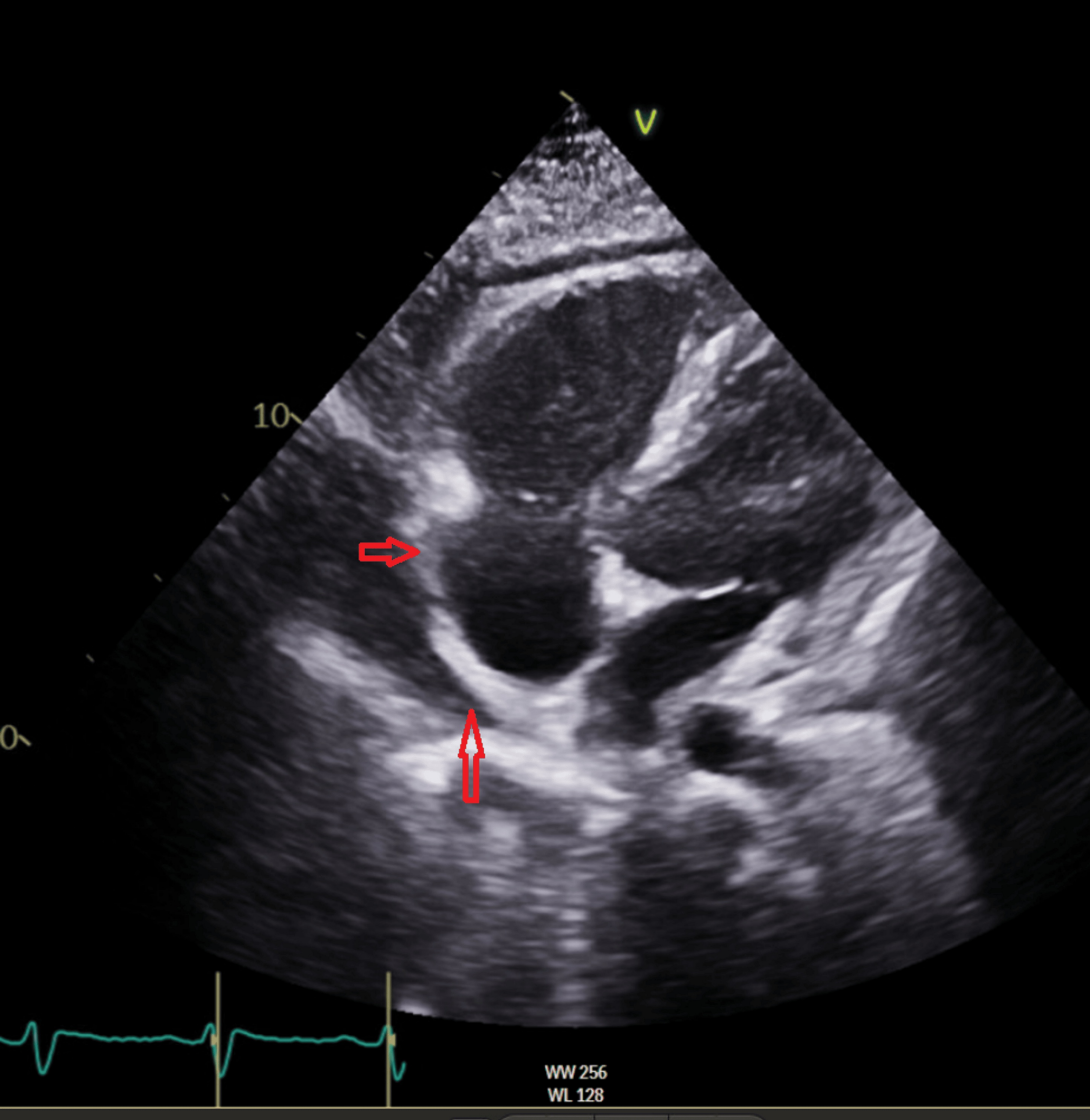
Transthoracic echocardiogram image showing a small to moderate pericardial effusion.

Bedside transthoracic echocardiogram (TTE) showed a large pericardial effusion with a diastolic measurement of 3.5 cm. Some components of right ventricular diastolic collapse and tricuspid valve (TV) inflow variation above 50% were noted. The inferior vena cava (IVC) was dilated but not collapsible, suggesting cardiac tamponade physiology. Cardiology performed emergent pericardiocentesis with pericardial drain placement and ibuprofen and colchicine were started for the treatment of acute pericarditis. TTE was performed on hospital day three to assess the pericardial effusion, and a significant improvement in the size of the pericardial effusion was noted (Figure 4).

The pericardial drain was removed after six days when bedside TTE showed resolution of the pericardial effusion. The patient’s symptoms improved significantly during the hospital course, and he was discharged home on colchicine 0.6 mg two times daily for three months and ibuprofen 600 mg three times daily (T.I.D.) for two weeks, followed by a weekly taper of 200 mg T.I.D. Clozapine remained held during hospitalization and was discontinued on discharge.

## Discussion

The exact mechanism by which clozapine causes cardiac side effects is unclear. Kilian et al. proposed an IgE-mediated hypersensitivity (type 1 allergic reaction) or type 3 allergic reaction (serum sickness) as the mechanism of clozapine-induced myocarditis based on findings of peripheral eosinophilia and eosinophilic inclusions on cardiac biopsy [8]. Abdel-Wahab et al. showed that pro-inflammatory cytokines, including tumor necrosis factor-alpha (TNF-a), increased in a dose-dependent fashion in rat models of clozapine-induced cardiotoxicity [9].

Clozapine treatment in most modern countries now requires a monitoring plan to ensure severe side effects are identified when they arise. The United States has adopted a mandatory registry-based strategy called Clozapine Risk Evaluation and Mitigation (REMS) to manage the risk of severe neutropenia associated with clozapine treatment [10]. Baseline absolute neutrophil count (ANC) levels are measured and trended as frequently as daily depending on subsequent ANC values following treatment initiation. While universally monitoring programs include specific guidelines focused on mitigating the risk of developing agranulocytosis, protocols for cardiac monitoring are usually not mandatory, lack consensus, and are uncommonly implemented [11,12]. Goldsmith et al. found that only 9% of surveyed providers in the United States reported using a cardiac screening protocol when initiating clozapine [13].

Proposed protocols for cardiac monitoring usually include obtaining baseline troponin (Tn), C-reactive protein (CRP), B-type natriuretic peptide (BNP), transthoracic echocardiogram (TTE), and/or electrocardiogram (EKG) before starting clozapine. The patient in our case had an EKG prior to starting clozapine but otherwise had no other baseline cardiac testing or subsequent monitoring. Various monitoring regimens have been proposed in the literature. Ronaldson et al. suggest obtaining baseline TTE and cardiac biomarkers (Tn, CRP) followed by repeat cardiac biomarkers on days 7, 14, 21, and 28 [14]. Griffin et al. suggest EKG and cardiac biomarkers (Tn, BNP, CRP) at baseline and weekly for eight weeks [15]. These protocols are designed to target myocarditis caused by clozapine since 80% of cases occur within four weeks of drug initiation and 90% within eight weeks [16].

The duration of these regimens would be insufficient for the patient in our case, as he was diagnosed with pericarditis four months after clozapine initiation, with cases having been observed in the literature developing seven days to seven years after drug initiation [17]. Our patient’s pattern of cardiac biomarkers on presentation was nonspecific, with just mildly elevated CRP levels along with negative troponin and BNP values, but EKG changes of low voltages concerning tamponade were present. Our patient’s presentation of pericarditis was further complicated by pericardial effusion, causing tamponade physiology that required urgent pericardial drain placement. TTE and chest CT were ultimately utilized to diagnose the patient’s effusion. We feel that given the life-threatening nature of cardiac side effects due to clozapine, it is imperative that all patients be placed on some kind of active cardiac monitoring protocol when starting treatment. The optimal testing protocol and duration of testing require more investigation to establish consensus guidelines. Providers must continue to have a high index of suspicion for cardiac side effects from clozapine, such as pericarditis, even in patients who have had cardiac screening and who have safely taken the drug for several years. Providers must also recognize that severe cardiac side effects beyond cardiomyopathy and myositis can occur, namely in the form of pericarditis and pericardial effusion with tamponade.

There have been several case reports of successful re-introduction of clozapine following episodes of clozapine-induced cardiac side effects [16]. Cook et al. have even proposed an intensive monitoring protocol for clozapine re-introduction that involves repeating EKG and TTE every two weeks, followed by monthly and repeating cardio biomarkers (Tn, CRP) twice a week, followed by monthly [16]. In patients with treatment-resistant schizophrenia (TRS) who lack efficacious alternatives to clozapine and who have a history of intolerance to clozapine due to cardiac side effects, the decision of whether to reintroduce clozapine must be individualized.

## Conclusions

Cardiovascular side effects of clozapine, including myocarditis, QTc prolongation, and rarely acute pericarditis, have been reported. However, acute pericarditis with a large pericardial effusion leading to life-threatening cardiac tamponade has only been reported in a handful of cases. Establishing a monitoring protocol like electrocardiogram, laboratory markers, and echocardiogram for patients on clozapine could prevent this potentially life-threatening cardiac complication and other serious side effects, minimizing morbidity and mortality and improving the overall safety of taking the medication. Further research is warranted to establish consensus guidelines on optimal cardiac monitoring protocols and their duration.
